# Implant replacement and anaplastic large cell lymphoma associated with breast implants: a quantitative analysis

**DOI:** 10.3389/fonc.2023.1202733

**Published:** 2023-10-19

**Authors:** Martina Vittorietti, Sergio Mazzola, Claudio Costantino, Daniele Domenico De Bella, Santo Fruscione, Nicole Bonaccorso, Martina Sciortino, Davide Costanza, Miriam Belluzzo, Alessandra Savatteri, Fabio Tramuto, Paolo Contiero, Giovanna Tagliabue, Palmira Immordino, Francesco Vitale, Arianna Di Napoli, Walter Mazzucco

**Affiliations:** ^1^ SEAS Department, University of Palermo, Palermo, Italy; ^2^ U.O.C. of Clinical Epidemiology with Cancer Registry, Azienda Ospedaliera Universitaria Policlinico di Palermo, Palermo, Italy; ^3^ PROMISE Department, University of Palermo, Palermo, Italy; ^4^ Environmental Epidemiology Unit, Fondazione IRCCS Istituto Nazionale dei Tumori, Milan, Italy; ^5^ Cancer Registry Unit, Fondazione IRCCS Istituto Nazionale dei Tumori, Milan, Italy; ^6^ Department of Clinical and Molecular Medicine, University of Rome “Sapienza”, Rome, Italy; ^7^ College of Medicine, University of Cincinnati, Cincinnati, OH, United States

**Keywords:** anaplastic large cell lymphoma associated with breast implants, breast implants replacement, epidemiology, time to disease onset, treatment outcomes, quantitative analysis

## Abstract

**Introduction:**

Breast implant-associated anaplastic large-cell lymphoma (BIA-ALCL) is a rare form of non-Hodgkin T-cell lymphoma associated with breast reconstruction post-mastectomy or cosmetic-additive mammoplasty. The increasing use of implants for cosmetic purposes is expected to lead to an increase in BIA-ALCL cases. This study investigated the main characteristics of the disease and the factors predicting BIA-ALCL onset in patients with and without an implant replacement.

**Methods:**

A quantitative analysis was performed by two independent researchers on cases extracted from 52 primary studies (case report, case series, and systematic review) published until April 2022 and searched in PubMed, Scopus, and Google-Scholar databases using “Breast-Implant” AND/OR “Associated” AND/OR “Anaplastic-Large-Cell-Lymphoma”. The statistical significance was verified by Student’s *t*-test for continuous variables, while Fisher’s exact test was applied for qualitative variables. Cox model with time-dependent covariates was used to estimate BIA-ALCL’s onset time. The Kaplan–Meier model allowed the estimation of the probability of survival after therapy according to breast implant exposure time.

**Results:**

Overall, 232 patients with BIA-ALCL were extracted. The mean age at diagnosis was 55 years old, with a mean time to disease onset from the first implant of 10.3 years. The hazard of developing BIA-ALCL in a shorter time resulted significantly higher for patients not having an implant replacement (hazard ratio = 0.03; 95%CI: 0.005–0.19; *p*-value < 0.01). Patients with implant replacement were significantly older than patients without previous replacement at diagnosis, having a median time to diagnosis since the first implant of 13 years (7 years in patients without replacement); anyway, the median time to BIA-ALCL occurrence since the last implantation was equal to 5 years.

**Discussion:**

Our findings suggest that, in BIA-ALCL patients, the implant substitution and/or capsulectomy may delay the disease’s onset. However, the risk of reoccurrence in an earlier time should be considered in these patients. Moreover, the time to BIA-ALCL onset slightly increased with age. Selection bias, lack of awareness, misdiagnosis, and limited data availability could be identified as limits of our study. An implant replacement should be considered according to a risk stratification approach to delay the BIA-ALCL occurrence in asymptomatic patients, although a stricter follow-up after the implant substitution should be recommended.

**Systematic Review Registration:**

https://www.crd.york.ac.uk/PROSPERO, identifier: CRD42023446726.

## 1 Introduction

Breast implant-associated anaplastic large-cell lymphoma (BIA-ALCL) is a non-Hodgkin T-cell lymphoma, occurring adjacent to a breast implant implanted for either reconstruction or cosmetic purposes, characterized by the proliferation of large pleomorphic tumor cells uniformly expressing CD30 and negative for anaplastic lymphoma kinase (ALK) protein (1). The first case of BIA-ALCL was reported in 1997, but it was recognized by the World Health Organization (WHO) as a provisional and then a definitive, nosological entity only in 2016 and 2022, respectively (2–4). The National Comprehensive Cancer Network established guidelines for standardized diagnosis and management, which were further updated in 2022 (5). Before 2016, similar to other non-Hodgkin lymphomas, BIA-ALCL patients were staged by the Ann Arbor classification, based on affected lymph node stations or extra-nodal sites and the presence of symptoms such as fever, weight loss, and sweating. Since BIA-ALCL could manifest as a neoplastic effusion localized to the space between the implant and the scar fibrotic capsule, or as an infiltrating tumor mass with or without lymph node and/or distant organ metastasis, starting from 2016 the commonly used staging system for BIA-ALCL became the MD Anderson TNM (6). BIA-ALCL is a rare cancer with an incidence rate that varies greatly across countries. However, the exact incidence or prevalence of BIA-ALCL cannot be easily estimated due to potential under-reporting and the lack of information about the total number of patients receiving a breast implant (7). According to data from the U.S. Food and Drug Administration (FDA), to date, 59 deaths out of 1,130 cases of BIA-ALCL have been reported worldwide, suggesting that a prompt diagnosis and an optimal treatment are effective in reducing disease mortality (6–9). Although data confirm the absolute rarity of the disease, due to the growing use of breast implants, it has been estimated that, in the near future, there could be a significant increase in BIA-ALCL cases. Interestingly, there are about 35 million women with breast implants worldwide, with about 450,000 new breast prostheses implanted every year in the US. In the US, the priority choice is round-shaped smooth breast implants, whereas in Europe approximately 85% of implants used were anatomical-shaped textured breast implants (8, 10). In 2011, the FDA issued an alert on the possible association between breast implant and BIA-ALCL, which allowed the deployment of a monitoring activity on the distribution of certain types of prosthetic implants (11). Later on, in 2019, the International Agency for Research on Cancer included the breast implants on the list of high-priority agents to be evaluated as a potential human carcinogenic agent (12). More recently, cases of ALCL associated with implants other than breast implant have been reported, potentially addressing a concurrent increase in the incidence of these neoplasms (13–15). Patients diagnosed with BIA-ALCL were mostly carriers of a textured implant, suggesting a moderate weight of evidence for a causal relationship between textured breast implants and ALCL (16). By contrast, to date, only 37 cases were reported in patients receiving smooth implants (7). However, of these 37 patients, eight had a history of at least one textured implant, one received a pure smooth implant, while in the remaining 28 the prior implant history was unknown (7). According to the available literature, the estimated latency time for BIA-ALCL occurrence since the implant implantation varied between less than a year to more than 20 years, but it remains uncertain which factors could influence this timing (17). To fill these gaps of knowledge, we performed a quantitative analysis using individual BIA-ALCL patient data extrapolated from available primary studies. We aimed to better describe the main characteristics of the disease according to age at time of diagnosis, time from the first and the last implant to diagnosis, reason for implant, type of implant (surface and fill), clinical presentation, type of treatment, and outcome. Furthermore, following the hypothesis that an implant replacement may play a role in pathogenesis and prognosis, we estimated any potential difference in the time to disease onset and in the treatment outcomes in BIA-ALCL patients who did or did not have breast implant replacement over time.

## 2 Materials and methods

The scientific literature was searched to identify primary studies on BIA-ALCL, published until April 2022, from which individual patient data was extrapolated.

At first, the Population, Exposure, Control, and Outcomes method was employed to define the research strategy that was conducted through PubMed, Scopus, and Google Scholar databases using the following research string: “Breast Implant” AND/OR “Associated” AND/OR “Anaplastic Large Cell Lymphoma” (18). Research and selection of the studies were carried out independently by two reviewers, in accordance with the Preferred Reporting Items for Systematic Reviews and Meta-Analyses statement, and a consensus was reached at the end. Studies reporting on an individual with base information of the characteristics of BIA-ALCL cases in patients who have or have not had breast implant replacements over time were included in the analyses. Data were extracted from each study by two researchers independently and then collected in an electronic database to perform the statistical analyses. The study was registered on PROSPERO international prospective register of systematic reviews (CRD42023446726).

The variables of interest collected from the primary studies and included in the dataset were as follows: age at diagnosis, time to onset from the first breast implant implantation, reason for the breast implantation (cosmetic or reconstructive), implant replacement (yes/no), breast implant type (texturized or smooth), implant fill (saline or silicone), time to onset from the last breast implant, number of implant substitutions, disease signs and/or symptoms, disease stage (TNM and Ann Arbor), type of treatment (implant removal, total capsulectomy, surgical intervention with radiotherapy, and/or chemotherapy), and outcomes at follow-up (disease-free survival, persistence of disease, death due to BIA-ALCL, and death due to other causes) (**Supplementary Table S1**).

### 2.1 Statistical analysis

A total of 232 cases of BIA-ALCL were extracted from the primary studies according to completeness of the variable of interest (1, 9, 19–85). A descriptive analysis was conducted on the characteristics of the sample obtained. Mean and median values, with standard deviations and ranges, for quantitative variables as well as absolute and relative frequencies for the categorical (qualitative) variables were calculated. Next, we performed a comparative statistical analysis by reason of implant (cosmetic and reconstructive) and implant replacement (at least one and none). To this end, we compared groups by median age at diagnosis, median onset time from the first breast implant implantation, clinical presentation at diagnosis, type of treatment, and clinical outcomes at follow-up. Statistical significance was verified by Student’s *t*-test for continuous variables, except for the onset time for which a non-parametric median test was used. Fisher’s exact test was applied for the qualitative variables.

The time to BIA-ALCL onset was estimated by applying the Cox model with time-dependent covariates. In the presence of longitudinal data, the Cox model with time-dependent covariates proved its superiority with respect to its time-independent counterpart (86). We included in the model the time elapsed between the first breast implantation and the BIA-ALCL onset as the dependent variable, while as independent variable we considered “reason for implantation (cosmetic or reconstructive)” and the time-dependent variables “implant replacement” and “age since implantation to time to disease onset”. We estimated the probability of not developing BIA-ALCL in patients with and without implant replacement (with age taken at the average value for both groups) and the hazard ratios (HRs) for every covariate in the model with their 95% confidence intervals (95%CIs).

To estimate the factors associated with outcomes at follow-up, we applied the Kaplan–Meier model of survival, conditioned to a previous breast replacement (87). More specifically, we explored the association between the survival time since the first breast implantation and the disease onset and the outcomes at follow-up (disease-free survival *versus* disease persistence and/or death from BIA-ALCL) and clinical presentation (seroma *versus* invasive) in association with the type of treatment (implant removal and/or total capsulectomy for seroma; surgery with radiation and/or chemotherapy for invasive presentation). To this end, since the cases were staged through different methods, we preferred to consider the clinical presentation of the disease (seroma *versus* invasive) as a proxy of early or advanced stages documented by the two available staging approaches (TNM and Ann Arbor). In brief, the Kaplan–Meier model allowed the estimation of the probability of survival after therapy according to breast implant exposure time. Any difference in the comparison between the survival curves was statically verified by applying the log rank test. The significance was set at a *p*-value <0.05. For the statistical analysis, R software was used–—in particular, the R package survival (19, 20).

## 3 Results

**Figure 1:**
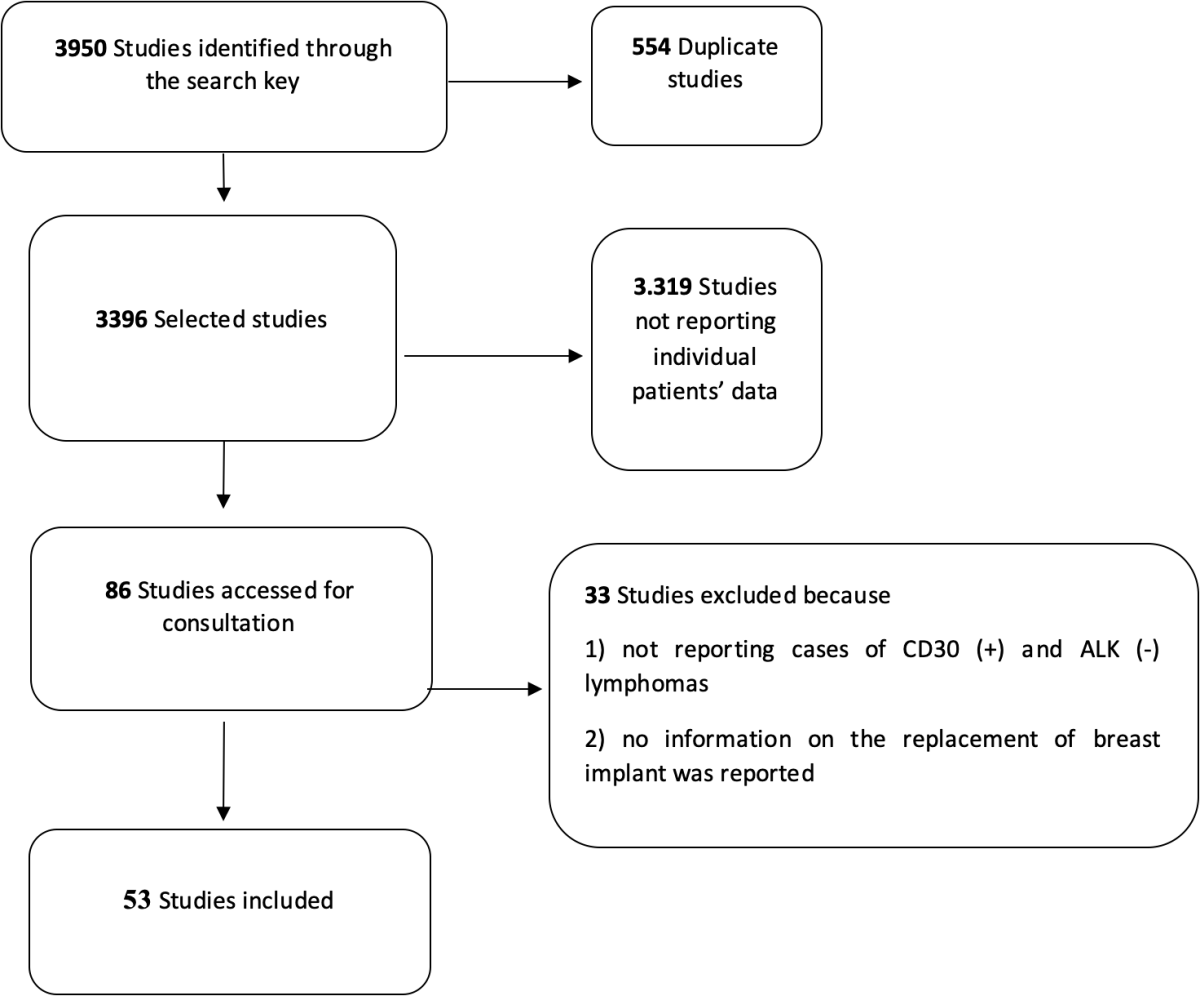
Flow diagram for the selection of the primary studies on breast implant-associated anaplastic large-cell lymphoma included in the quantitative analysis.

The initial search allowed the identification of 3,950 studies on BIA-ALCL (**Figure 1**). After eliminating duplicates and articles that did not match the inclusion criteria, 86 studies were selected and consulted. A total of 33 other studies were further excluded because they did not include relevant information such as implant substitution or the associated immunophenotype of tumor cells (i.e., CD30 and ALK protein expression). Finally, 53 articles were considered for the analyses (**Supplementary Table S2**).

Regarding the characteristics of the 232 patients diagnosed with BIA-ALCL included in the quantitative analysis (data not shown), a mean age at diagnosis of 55 years old (SD 11.8; median age: 55 years old; range: 29 to 87) was documented. The mean time of BIA-ALCL onset from first implant was 10.3 years (SD 5.8), while the median time was 9 years, with a range of 1 to 32 years.

According to the reason of implant, 41.0% of the cases were related to cosmetic purposes and 45.0% to reconstructive reasons, while for 14.0% of the cases this data was not available (**Table 1**). The BIA-ALCL patients receiving breast implantation for cosmetic purposes at diagnosis resulted in significantly younger women having an oncological post-mastectomy breast reconstruction (mean age at diagnosis: 50 *versus* 60 years old; *p*-value: <0.0001), but no statistically significant difference was highlighted for the median time to BIA-ALCL occurrence since the first breast implantation between the two groups (*p*-value: 0.31) (**Table 1**).

**Table 1:** Comparison of the characteristics of breast implant-associated anaplastic large-cell lymphoma (BIA-ALCL) cases by reasons of implant and implant replacement.

		n. (%)	Mean age (years old) at diagnosis	*p*-value	Median time (in years) to BIA-ALCL occurrence since the first implantation (range)	*p*-value
Reason of implant	Cosmetic	95 (46)	50	<0.0001	7 (1–32)	0.31
	Reconstructive	104 (47)	60		7 (1–31)	
	Not available	33 (15)	–		–	
Breast implant replacement	At least one	84 (48)	57	<0.05	13 (4–32)	<0.0001
	None	86 (49)	52		7 (1–30)	
	Not available	62 (19)	–		–	

An implant replacement was documented in 36.0% of the cases, while 37.0% of the patients did not undergo surgery for replacement; for the remaining 27% of the cases, data on replacement were not available (**Table 1**). Compared to patients who did not substitute their implant, the BIA-ALCL patients who received at least a breast implant replacement were significantly older (mean age at diagnosis: 57 *versus* 52 years old; *p*-value: <0.05) and showed a longer median time to diagnosis since the first breast implantation [13 years (range: 4–32) *versus* 7 years (range: 1–30); *p*-value: <0.0001] (**Table 1**). Moreover, the median time to BIA-ALCL occurrence since the last implantation was equal to 5 years (range: 0.3–10), although this information was available only for 23 of 84 patients (data not shown).

Breast implants with a texturized surface were the most commonly used (136/232, 59.0%; in particular, 70/136 were macro-texturized, 3/136 micro-texturized, and 63/136 were not specified), with a single BIA-ALCL case (0.4%) reporting the use of a smooth surface. In 41.0% of cases, the information on breast implant surface was not available (data not shown). The most frequent implant fill was silicon (99/232, 43.0%), followed by saline solution (31/232, 13.0%), with a saline/silicon filled breast implant reported in a single BIA-ALCL case (0.4%), while for the remaining 44.0% of cases this data was not available (data not showed).

Of the 82 BIA-ALCL cases staged at diagnosis with Ann Arbor classification, 60 cases (73.2%) had tumor stage I and IE, while more advanced stages were less frequent (22 cases, 14.0%). Similarly, most of the 102 patients staged, according to the MD Anderson TNM classification, were diagnosed at an early stage; in particular, 77 (75.5%) had tumor stages I, IA, IB, and or IC and 12 cases (11.8%) were referred to tumor stage IIA, while just 13 cases (12.7%) presented the most advanced stages IIB, III, or IV.

**Figure 2:**
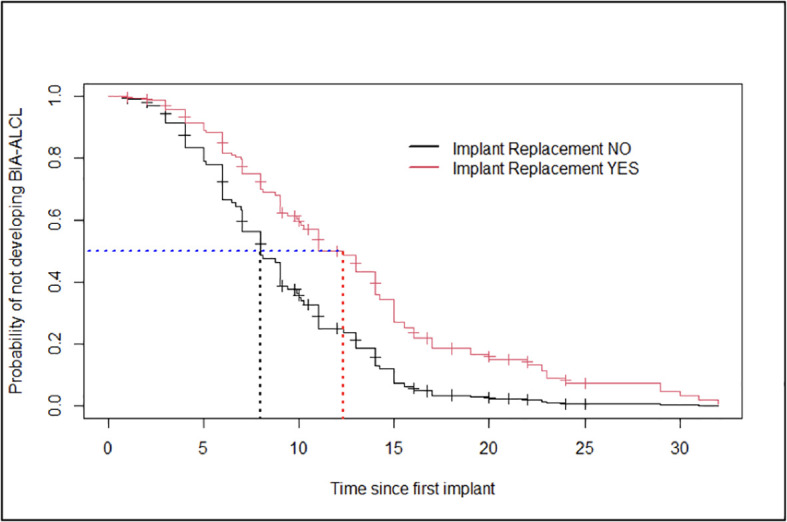
Estimates of the Cox model’s probability of not developing breast implant-associated anaplastic large-cell lymphoma for patients who had breast implant replacement and patients who did not have any (age taken at the average value for both groups).

No statistically significant difference (*p*-value: 0.157) was reported in the clinical presentation at diagnosis suggestive of a confined (i.e., seroma) *versus* an invasive disease (i.e., tumor mass and/or lymphadenopathy) in patients who underwent a previous breast implant replacement as compared to patients who did not (data not shown). However, patients having at least an implant replacement showed 50.0% probability of developing BI-ALCL in 12 years (95%CI: 10.0–15), while patients who did not have implant replacement had a similar probability in a significantly shorter time of 8 years (95%CI: 7.0–9.8) (**Figure 2**).

**Table 2** shows the results of the Cox model in terms of hazard ratios. The hazard of developing BIA-ALCL in a shorter time resulted significantly higher for patients not having an implant replacement (HR = 0.03; 95%CI: 0.005–0.19; *p*-value = <0.01). In other words, as the number of implant replacements increased, the time to the BIA-ALCL onset became longer than in patients who had not replaced the implant in the past.

**Table 2:** Cox model with time-dependent covariates and estimated hazard ratios.

Covariates	Hazard ratio (95%CI)	*p*-value
Implant replacement (*t*)	0.03 (0.005–0.19)	<0.01
Age (*t*)	0.95 (0.93–0.97)	<0.0001
Implant replacement (*t*): age (*t*)	1.05 (1.02–1.08)	<0.001

*(t)* stands for time expressed in years. Implant replacement *(t)* stands for time by first implant replacement expressed in years. Age *(t)* stands for patients age expressed in years.

Furthermore, the hazard of developing BIA-ALCL in a shorter time resulted slightly lower for patients with a higher age (HR = 0.95; 95%CI: 0.93–0.97; *p*-value <0.0001).

However, a significant interaction between the time to implant replacements and patient’s age was highlighted, with older patients having an implant replacement developing BIA-ALCL in a later time (HR = 1.05: 95%CI: 1.02–1.08; *p*-value <0.001). The covariate “reason for implantation” was not included in the model as it resulted to be not significant (HR = 0.63; 95%CI: 0.09–4.44; *p*-value = 0.33).

**Figure 3:**
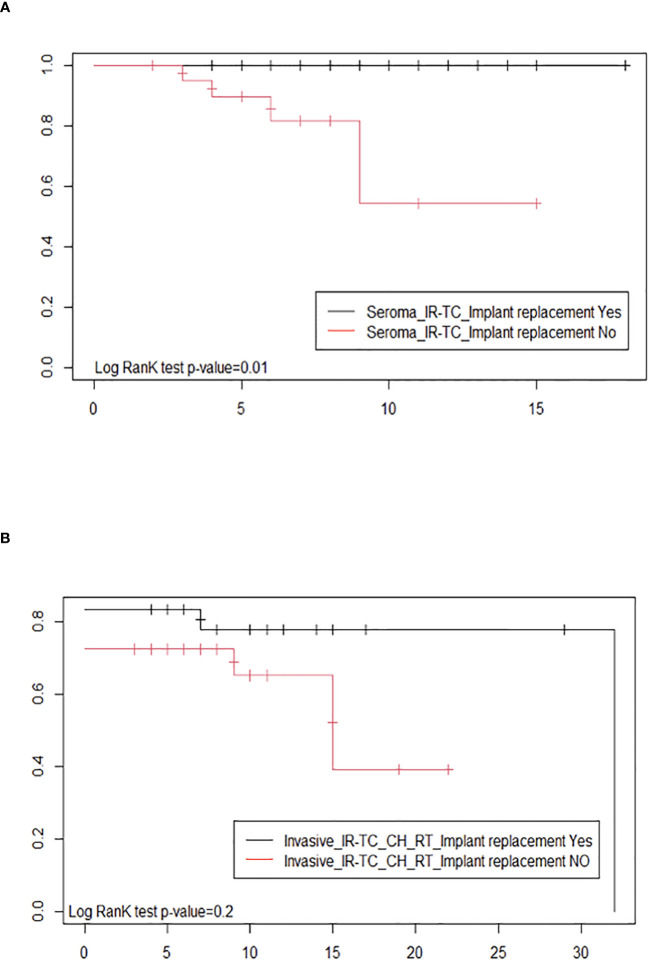
Estimates of the probability of survival after therapy by Kaplan–Meier survival curves of breast implant-associated anaplastic large-cell lymphoma patients with and without implant replacement: **(A)** with seroma clinical presentation, undergoing surgery only (implant removal and total capsulectomy) and **(B)** with a tumor mass or lymph node involvement and who, in addition to surgery (implant removal and total capsulectomy), were treated with chemotherapy and/or radiation therapy.

Due to the lack of homogeneity in the staging system, we used the type of clinical presentation (seroma *versus* tumor mass and/or lymphadenopathy) and the type of treatment (implant removal with total capsulectomy *versus* surgery followed by chemotherapy and/or radiotherapy) as a proxy of the tumor stage to compare the probability of survival for BIA-ALCL patients who received implant replacement with respect to those who did not (**Figure 3**). The probability of a disease-free treatment outcome in BIA-ALCL patients with seroma clinical presentation, who underwent surgery only (implant removal and total capsulectomy; 45 cases), resulted significantly higher (*p*-value: 0.01) in patients who had an implant replacement in the past than in patients who did not have one (**Figure 3A**). The Kaplan–Meier curves were not significantly different (*p*-value: 0.2) in BIA-ALCL patients with a more invasive tumor, who underwent surgery in association with chemotherapy and/or radiation therapy (54 cases, 12 of which were lost to follow-up), according to a previous implant replacement (**Figure 3B**). These results highlight that, according to the same clinical presentation, the treatments resulted effective in improving the probability of survival only in patients with an early-stage disease who underwent a previous implant replacement with respect to those who did not undergo implant replacement in the past.

## 4 Discussion

As most of the available evidence on BIA-ALCL, a rare cancer affecting patients undergoing post-mastectomy breast reconstruction or cosmetic additive mammoplasty, deal with case reports and a few observational studies, we conducted a quantitative analysis on individual patients’ data extrapolated from primary published studies to improve the sample size and, therefore, the existing gap of knowledge (9, 21–23). This approach allowed us to investigate, at the same time, factors potentially predicting the clinical presentation and outcome of the disease.

As expected, in our series, BIA-ALCL patients receiving cosmetic mammoplasty resulted to be significantly younger at diagnosis than patients having a breast implant for post-mastectomy breast reconstruction. However, no significant difference in the median time to disease onset since the first implantation or in the hazard of developing the BIA-ALCL was found. This data may reflect the evidence that the time of exposure to the implant, regardless of the age at implantation and any possible condition associated with the underlying disease, may play a role in favoring BIA-ALCL development. Therefore, individuals undergoing breast surgery for cosmetic reasons since younger ages seem to have a similar risk in terms of median time to BIA-ALCL onset of patients receiving a breast implant for oncologic reasons. In the same direction, we did not find any difference in the clinical presentation (considered as a proxy of the disease staging) of BIA-ALCL in patients undergoing breast implantation for cosmetic or reconstructive reasons. These findings taken together should recall the attention of researchers and epidemiologists according to the documented increasing use of implants for cosmetic purposes that might hamper the incidence of BIA-ALCL cases over time (10, 88). The current reported risk of BIA-ALCL is estimated to be one per 12,832, a striking increase from initial estimates of one per million (1, 89, 90). The estimated incidence has a significant variance worldwide, with European, Asian, and South American countries having the lowest relative incidences and Australia and New Zealand reporting the highest rates (9).

Interestingly, both the age at diagnosis and the time of disease onset since the first breast implantation were significantly higher in BIA-ALCL patients who underwent at least one implant replacement over time, as compared with those who did not; however, after implant substitution, the risk of reoccurrence in an earlier time should be considered in BIA-ALCL patients. Moreover, the estimated probability of developing BIA-ALCL in a longer time was significantly higher for patients who had at least a previous breast implant replacement as compared to patients who did not have any in the past. In the same direction, the hazard of developing BIA-ALCL for patients who had at least an implant replacement was 1/30 lower with respect to patients who did not, and as the number of implant replacements increased, the time to the onset of BIA-ALCL became longer than in patients who did not undergo surgery in the past to replace the implant. This body of evidence may suggest a potential role in the delay of the disease onset played by the implant substitution and/or by the possible related capsulectomy. Complete capsulectomy with clear margins have been reported to be the most curative treatment in patients with early-stage BIA-ALCL (6, 9). On the contrary, incomplete capsulectomy with or without systemic treatment or re-implantation was found to be followed by disease persistence/recurrence (6, 9). In three patients, complete remission was achieved upon implant removal and/or surgical removal of the residual fibrous capsule (24, 25, 91). Interestingly, in the case series of Lamaris GA et al., reconstruction with smooth implants was either performed immediately or delayed from BIA-ALCL diagnosis in 13 out of 18 patients, and all of them were in complete remission, therefore suggesting the use of smooth surfaces for implant replacement (92). Unfortunately, it was not always possible to find information about the completeness of capsulectomy in previous implant replacement surgeries, thus limiting our analyses. More specifically, in our series, we do not have accurate data on the reason of implant removal and on the histopathology of the capsules removed; hence, we cannot exclude that BIA-ALCL was already present at the time of first explant. In the report from Keith L et al., a patient with bilateral implants but monolateral recurrent seromas and receiving multiple revisions over a period of 13 years was finally diagnosed with BIA-ALCL on the same side of the recurrent seroma when capsulectomy with histopathological examination was performed (93). In one of the previously reported cases, the patient was diagnosed with BIA-ALCL after 14 years from first implantation and 4 months only after implant revision and incomplete capsulectomy made for seroma due to suspected intracapsular breast implant rupture (25). These cases suggest that the disease begins and persists on the side of the seroma and that the lack of adequate pathological examination of the fluid, together with the incomplete capsulectomy performed, had likely delayed the BIA-ALCL diagnosis. Moreover, according to the implant package leaflet, patients who had their implants replaced had an increased risk of future complications compared to those who had first time (primary) reconstruction (26). Concordantly, we found that patients with implant replacement developed BIA-ALCL from the last implant within a shorter median time compared to patients who did not undergo implant revision (5 years vs. 7 years). Nevertheless, this data was available only for a small number of patients, thus not allowing any conclusions to be drawn. In BIA-ALCL pathogenesis, the chronic inflammation elicited by the implant and the bacteria biofilm growing on its surface is thought to play a major role in favoring chromosomal instability, dysregulation of the epigenetic machinery, and mutations of genes of the JAK-STAT3 signaling responsible for the transformation of normal lymphocytes into lymphoma cells (22, 27, 28, 94, 95). However, more data on the reasons leading to implant revision (implant rupture, capsular contracture, implant displacement, recurrent seromas, and changing implant size) are needed to investigate the possible retarding effect of surgical implant substitution/capsulectomy on BIA-ALCL development. We do not have any direct proof that the first implant was involved in the development of BIA-ALCL. However, the shortest time to BIA-ALCL onset after the second implantation was 0.3 years from our findings and 0 years from the data reported by the FDA (7). Considering that lymphomagenesis requires multiple molecular alterations and even in patients with genetic predisposition BIA-ALCL occurred after 3, 7, and 19 years from implantation, respectively, the time to BIA-ALCL onset in some of the patients with implant replacement seems to be too short (29, 30, 96). Furthermore, according to our knowledge, to date, no one case of BIA-ALCL developed in a patient with a “pure” history of smooth surface implants, suggesting that the previous textured implant/expander could have played a role in lymphomagenesis (7, 97). This body of evidence, taken together with our findings, may support a prominent role played by the first implant.

In keeping with this, it would be of interest to include data on the reason of implant revision, radicality of capsulectomy, and the time from first implantation among the variables considered by the FDA in the statistics on global medical device reports (7) to better clarify the possible contribution of the type of complication, surgical procedures, and residual fibrous capsule disease in the mutagenic effect of chronic inflammation on immune cells (31).

Regarding the role of a patient’s age in the progression from the time of first implantation to the time to disease onset, it is interesting to highlight how in our quantitative analysis the time to BIA-ALCL onset slightly increased with age. Anyway, the documented interaction between age and implant replacement may suggest that, among patients having a previous implant replacement, the oldest ones had a slightly higher risk to develop BIA-ALCL. This result could be linked to the somatic mutation theory of aging based on which virtually all tissues accumulate somatic DNA mutations over time (98). If one of these mutations imparts a fitness advantage to the cell, this will clonally expand. The clonal outgrowth of hematopoietic cells (clonal hematopoiesis) is highly prevalent in the elderly and predisposes to hematological malignancies including T-cell lymphomas (99). In keeping with this, in a large proportion of BIA-ALCL patients, mutations in genes behaving as epigenetic modifiers such as KMT2C, KMT2D, and DNMT3A, already known to be involved in clonal hematopoiesis, have been documented (22, 27).

BIA-ALCL is a rare but potentially serious neoplastic condition, whose clinical presentation could be a seroma (a collection of fluid around the implant) or, less frequently, a tumor mass infiltrating normal tissues adjacent to the implant (7). It is still unclear whether the mass represents the evolution of the seroma or a distinct disease (6, 100). The seroma may be predictive of a better prognosis when associated with a locally confined disease timely diagnosed and treated. Our quantitative analysis showed no difference in the clinical presentation at diagnosis according to a prior breast implant replacement. However, the Kaplan–Meier model allowed the estimation that, among BIA-ALCL patients with a clinical seroma presentation who were surgically treated, those receiving a previous breast implant replacement had a better prognosis than those who did not undergo a previous breast implant substitution. Conversely, no difference in terms of prognosis was highlighted among BIA-ALCL patients with advanced diseases treated with surgery and radio and/or chemotherapy according to the breast implant replacement. This finding may indicate the effective role of local and/or systemic therapy in controlling the disease.

To date, the available literature supports a favorable prognosis if a complete surgical excision is performed both in early- and advanced-stage patients (6, 9). In keeping with this, BIA-ALCL relapses have been reported after incomplete or non-*en bloc* capsulectomy (21, 24, 101).

However, no data on the possible role of previous implant substitution and related capsulectomy to prevent BIA-ALCL relapse was provided. Our findings documented that, according to the same clinical presentation and surgical treatment, patients who underwent implant replacement had a better probability over time of a disease-free prognosis than patients who did not receive an implant replacement, while for BIA-ALCL patients with an invasive clinical presentation, the probability of a disease-free prognosis resulted to be not influenced by a previous implant replacement. However, as the absence of disease recurrence in this series of BIA-ALCL cases was based mainly on an immediate post-treatment assessment (22 months, range: 9–60). This quantitative analysis should be repeated on a longer follow-up period.

Notably, the 232 cases included in this study represent about 25% of the entire worldwide case series available from specialized clinical registries at the time of the literature search, with similar average age at diagnosis, time to disease onset since the first breast implant, and reason of implantation (8, 102). However, this quantitative analysis has some limitations that need to be discussed. First of all, a selection bias may have occurred according to the incompleteness of data extracted from primary studies or to the lack of variables that lead to the exclusion of some studies from the quantitative analysis. Moreover, the reporting bias of the different studies included in the quantitative analysis might have caused an unmeasurable heterogeneity.

Certainly, a future linkage between tumor registries and registries collecting information on implants could provide more affordable data to obtain a clear epidemiological picture of BIA-ALCL, which could have been underestimated according to the lack of awareness and misdiagnosis (8, 23, 32–34, 103). In our sample population of BIA-ALCL cases, we were not always able to obtain complete data on the prior implant placement time, neither on the reason for replacing the implants or the time since the patient has received the new implant. Information on the surgical procedure (*en bloc* capsulectomy or preservation of the fibrous capsule) was also frequently missing, thus limiting our quantitative analysis. To date, therefore, a clear picture of the risk factors associated with this disease is still missing, mainly because of nonspecific symptoms and delay to onset, poor awareness of the disease from both patients and surgeons (especially in the past years), and limited data availability (35). In women with breast implants, silicone particles have been isolated not only within the fibrous capsule but also from the tissues outside the capsule, indicating silicone gel bleeding and migration (36). Interestingly, BIA-ALCL was reported with synchronous contralateral silicone-induced granuloma of breast implant capsule as a possible different immune response to local immune reactions to silicone particles (37). Although the pathobiology of BIA-ALCL appears to be multifactorial, significant gaps in knowledge allow us to only speculate on the possible role of individual endogenous factors (i.e., genetic predisposition) and of specific exogenous factors (i.e., implant’s characteristics), thus limiting more precise epidemiologic studies (23).

Another limitation refers to the case-to-case design implemented in this study. Particularly, when applying the Cox model with time-dependent covariates to model the time of BIA-ALCL onset, as all the patients in the study have experienced the event, the interpretation of the probability of developing the event is challenging. Therefore, case–control studies comparing cases to patients receiving a breast implant, but not having developed BIA-ALCL, will allow for the obtainment of more reliable estimates.

Lastly, because of the use of different tumor staging systems, we used the type of clinical presentation and the type of treatment as surrogates of the stage. This alternative approach together with the high number of cases lost at follow-up could have influenced the Kaplan–Meier survival curves.

Despite the abovementioned limitations, we believe that our data may add knowledge on BIA-ALCL, contributing to underline the possible delayed onset of the disease. More specifically, in line with a recent study following a similar methodological approach, we can conclude that an implant replacement should be considered according to a risk stratification approach to delay any BIA-ALCL occurrence in asymptomatic patients (38), although a stricter follow-up after the implant substitution should be recommended as well in these patients. Last but not least, as the BIA-ALCL incidence rates may become higher than the one estimated in the past, in the age of digital health interoperability, the opportunity of a linkage between registries collecting data on all types of prosthetic implants and population-based cancer registries should be explored to better estimate the epidemiological impact over time of malignancies (ALCL, B-cell lymphomas, or solid tumors) occurring in association with breast and non-breast implants implanted both for curative and aesthetic purposes (13–15, 23, 39, 40, 102, 103).

## Data availability statement

The original contributions presented in the study are included in the article/**Supplementary Material**. Further inquiries can be directed to the corresponding author.

## Author contributions

WM, MV, and SM contributed to conception and design of the study. MV, SM, DDB, and DC organized the database. MV and SM performed the statistical analysis. WM, SM, and MV wrote the first draft of the manuscript. ADN and SF wrote sections of the manuscript. All authors contributed to the article and approved the submitted version.
