# Supplementary material for: Implant replacement and anaplastic large cell lymphoma associated with breast implants: a quantitative analysis
Source: Front Oncol. 2023 Oct 19;13:1202733. doi: 10.3389/fonc.2023.1202733 (PMC10622658; doi:10.3389/fonc.2023.1202733)
Supplement: Supplementary file 1 [file Table_1.docx]

Supplementary Material

Implant replacement and Anaplastic Large Cell Lymphoma Associated with Breast Implants: a systematic review and quantitative analysis.

**Martina Vittorietti, Sergio Mazzola, Claudio Costantino, Daniele Domenico De Bella, Santo Fruscione, Nicole Bonaccorso, Martina Sciortino, Davide Costanza, Miriam Belluzzo, Alessandra Savatteri, Fabio Tramuto, Paolo Contiero, Giovanna Tagliabue, Palmira Immordino, Francesco Vitale, Arianna Di Napoli, Walter Mazzucco**

*** Correspondence:** Santo Fruscione: santo.fruscione@unipa.it

# Supplementary Figures and Tables

**Supplementary Tab 1.** Variables included in the quantitative analysis.

| **Study** | **Year of publication** | **Age at diagnosis** | **Reason for the breast implantation** | | **Implant replacement** | | | **Breast implant type** | **Time from first implant** | | **Time to sub replacement** | | **Time from last implant** | | **Symptom** | | **Disease Stage** | | **Type of treatment** | | **Follow up** | | **Completeness** | |  |
| --- | --- | --- | --- | --- | --- | --- | --- | --- | --- | --- | --- | --- | --- | --- | --- | --- | --- | --- | --- | --- | --- | --- | --- | --- | --- |
| **Gaudet et al.** [52] | 2002 | x | | x | | x | x | | | x | | - | | x | | x | | x | | x | | - | | 9 | |
| **Sahoo et al.** [53] | 2003 | x | | x | | x | x | | | x | | x | | x | | x | | x | | x | | x | | 11 | |
| **Fritzsche et al.** [54] | 2006 | x | | x | | x | x | | | x | | - | | x | | x | | x | | x | | - | | 9 | |
| **Olack et al.** [55] | 2006 | x | | x | | x | x | | | x | | - | | x | | x | | x | | x | | x | | 10 | |
| **De Jong et al.** [56] | 2008 | x | | - | | x | x | | | x | | x | | x | | - | | x | | - | | - | | 7 | |
| **Roden et al.** [57] | 2008 | x | | x | | x | x | | | x | | - | | x | | x | | x | | x | | x | | 10 | |
| **Wang et al.** [58] | 2008 | x | | x | | x | x | | | x | | x | | x | | x | | - | | x | | x | | 10 | |
| **Alobeid et al.** [59] | 2009 | x | | x | | x | x | | | x | | - | | x | | x | | x | | x | | - | | 9 | |
| **Bishara et al.** [60] | 2009 | x | | x | | x | x | | | x | | - | | x | | x | | x | | x | | x | | 10 | |
| **Farkash et al.** [61] | 2009 | x | | x | | x | x | | | x | | - | | x | | x | | x | | x | | x | | 10 | |
| **Li & Lee et al.** [62] | 2009 | x | | x | | x | x | | | x | | - | | x | | x | | - | | x | | x | | 9 | |
| **Miranda et al.** [63] | 2009 | x | | x | | x | x | | | x | | - | | x | | x | | x | | x | | x | | 10 | |
| **Do et al.** [64] | 2010 | x | | x | | x | x | | | x | | - | | x | | x | | x | | x | | - | | 9 | |
| **Carty et al.** [65] | 2011 | x | | x | | x | x | | | x | | - | | x | | x | | x | | x | | x | | 10 | |
| **Popplewell et al.** [66] | 2011 | x | | x | | x | x | | | x | | - | | x | | x | | x | | x | | x | | 10 | |
| **Aladily et al.** [67] | 2012 | x | | x | | x | x | | | x | | x | | x | | x | | x | | x | | x | | 11 | |
| **Smith et al.** [68] | 2012 | x | | x | | x | x | | | x | | - | | x | | x | | x | | x | | x | | 10 | |
| **Taylor et al.** [69] | 2012 | x | | x | | x | x | | | x | | x | | x | | x | | x | | x | | x | | 11 | |
| **Bautista-Quach et al.** [70] | 2013 | x | | x | | x | x | | | x | | - | | x | | x | | x | | x | | x | | 10 | |
| **De Silva et al.** [71] | 2013 | x | | x | | x | x | | | x | | x | | x | | x | | - | | x | | - | | 9 | |
| **Farace et al.** [72] | 2013 | x | | x | | x | x | | | x | | - | | - | | x | | x | | x | | x | | 9 | |
| **George et al.** [73] | 2013 | x | | x | | x | x | | | x | | - | | - | | x | | x | | x | | x | | 9 | |
| **Ivaldi et al.** [74] | 2013 | x | | x | | x | x | | | x | | x | | x | | x | | x | | x | | x | | 11 | |
| **Parthasarathy et al.** [75] | 2013 | x | | x | | x | x | | | x | | - | | x | | x | | x | | x | | x | | 10 | |
| **Weathers et al.** [76] | 2013 | x | | x | | x | x | | | x | | - | | - | | x | | x | | x | | - | | 8 | |
| **Zakhary et al.** [77] | 2013 | x | | x | | x | x | | | x | | - | | - | | x | | x | | x | | - | | 8 | |
| **Laurent et al.** [36] | 2015 | x | | x | | x | x | | | x | | - | | x | | x | | x | | x | | x | | 10 | |
| **Di Napoli et al.** [31] | 2016 | x | | x | | x | x | | | x | | x | | x | | x | | x | | x | | x | | 11 | |
| **Torres-Rivero et al.** [78] | 2016 | x | | x | | x | x | | | x | | - | | x | | x | | - | | x | | x | | 9 | |
| **Wong et al.** [79] | 2016 | x | | x | | x | x | | | x | | x | | x | | x | | x | | x | | - | | 10 | |
| **Alderuccio et al.** [80] | 2017 | x | | x | | x | x | | | x | | - | | x | | x | | x | | x | | x | | 10 | |
| **Garcia et al.** [81] | 2017 | x | | x | | x | x | | | x | | - | | x | | x | | x | | x | | - | | 9 | |
| **Johnson et al.** [82] | 2017 | - | | x | | x | x | | | x | | x | | x | | x | | x | | x | | x | | 10 | |
| **Nogueira Batista et al.** [83] | 2017 | x | | x | | x | x | | | x | | - | | x | | x | | - | | x | | x | | 9 | |
| **Patzelt** **et al.** [84] | 2017 | x | | x | | x | x | | | x | | - | | x | | x | | x | | x | | x | | 10 | |
| **Dashevsky et al.** [85] | 2018 | x | | x | | x | x | | | x | | x | | x | | x | | x | | x | | - | | 10 | |
| **Kricheldorff et al.** [86] | 2018 | - | | x | | x | x | | | x | | x | | x | | x | | x | | x | | x | | 10 | |
| **Ronchi et al.** [87] | 2018 | x | | x | | x | x | | | x | | - | | x | | x | | - | | x | | x | | 9 | |
| **Becherer et al.** [41] | 2019 | - | | x | | x | x | | | - | | - | | - | | x | | x | | x | | - | | 6 | |
| **D’Alessandris et al.** [88] | 2019 | x | | x | | x | x | | | x | | - | | x | | x | | x | | x | | x | | 10 | |
| **Fricke et al.** [89] | 2019 | x | | x | | x | x | | | x | | - | | x | | x | | - | | x | | - | | 8 | |
| **Har-Shai et al.** [43] | 2019 | x | | x | | x | x | | | x | | - | | x | | x | | - | | x | | x | | 9 | |
| **Campanale et al.** [9] | 2020 | x | | x | | x | x | | | - | | - | | x | | x | | x | | x | | x | | 9 | |
| **Evans et al.** [90] | 2020 | x | | x | | x | x | | | x | | - | | x | | x | | - | | x | | x | | 9 | |
| **Kim et al.** [91] | 2020 | x | | x | | x | x | | | x | | - | | x | | x | | x | | x | | x | | 10 | |
| **Laurent et al.** [24] | 2020 | x | | x | | x | x | | | x | | - | | x | | - | | x | | x | | x | | 9 | |
| **Lee et al.** [92] | 2020 | x | | x | | x | x | | | x | | - | | x | | x | | x | | x | | - | | 9 | |
| **Misad et al.** [93] | 2020 | x | | x | | x | x | | | x | | - | | x | | x | | - | | x | | - | | 8 | |
| **Nelson et al.** [25] | 2020 | x | | - | | x | x | | | x | | - | | x | | x | | - | | x | | - | | 7 | |
| **Ohishi et al.** [38] | 2020 | x | | x | | x | x | | | x | | - | | x | | x | | x | | x | | x | | 10 | |
| **Thienpaitoon et al.** [94] | 2020 | x | | x | | x | x | | | x | | - | | x | | x | | x | | x | | x | | 10 | |
| **Everest et al.** [1] | 2021 | x | | x | | x | x | | | - | | - | | - | | x | | x | | x | | - | | 7 | |
| **Barnea et al.** [23] | 2022 | x | | x | | x | x | | | x | | x | | x | | x | | x | | x | | x | | 11 | |

**Supplementary Table 2.** List of the 53 articles included in the study.

| **AUTORS** | **YEARS OF PUBLICATION** | **TYPE OF STUDY** | **N. CASES** |
| --- | --- | --- | --- |
| **Gaudet et al.** [50] | 2002 | CASE REPORT | 1 |
| **Sahoo et al.** [51] | 2003 | CASE REPORT | 1 |
| **Fritzsche et al.** [52] | 2006 | CASE REPORT | 1 |
| **Olack et al.** [53] | 2006 | CASE REPORT | 2 |
| **De Jong et al.** [54] | 2008 | CASE CONTROL | 5 |
| **Roden et al.** [55] | 2008 | CASE SERIES | 4 |
| **Wong et al.** [56] | 2008 | CASE REPORT | 1 |
| **Alobeid et al.** [57] | 2009 | CASE REPORT | 1 |
| **Bishara et al.** [58] | 2009 | CASE REPORT | 1 |
| **Farkash et al.** [59] | 2009 | CASE REPORT | 1 |
| **Li & Lee et al.** [60] | 2009 | CASE REPORT | 1 |
| **Miranda et al.** [61] | 2009 | SYSTEMATIC REVIEW | 3 |
| **Do et al.**  [62] | 2010 | CASE REPORT | 1 |
| **Carty et al.** [63] | 2011 | CASE REPORT | 1 |
| **Popplewell et al.** [64] | 2011 | SYSTEMATIC REVIEW | 7 |
| **Aladily et al.** [65] | 2012 | CASE REPORT | 13 |
| **Smith et al.** [66] | 2012 | CASE REPORT | 1 |
| **Taylor et al.** [67] | 2012 | CASE REPORT | 5 |
| **Bautista-Quach et al.** [68] | 2013 | CASE REPORT | 1 |
| **De Silva et al.** [69] | 2013 | CASE REPORT | 1 |
| **Farace** **et al.** [70] | 2013 | CASE REPORT | 1 |
| **George et al.** [71] | 2013 | CASE REPORT | 1 |
| **Ivaldi et al.** [72] | 2013 | CASE REPORT | 2 |
| **Parthasarathy** **et al.** [73] | 2013 | CASE REPORT | 1 |
| **Weathers et al.** [74] | 2013 | CASE REPORT | 1 |
| **Zakhary et al.** [75] | 2013 | CASE REPORT | 1 |
| **Laurent et al.** [34] | 2015 | SYSTEMATIC REVIEW | 5 |
| **Di Napoli et al.** [29] | 2016 | CASE SERIES | 7 |
| **Torres-Rivero et al.** [76] | 2016 | CASE REPORT | 1 |
| **Wang et al.** [77] | 2016 | CASE REPORT | 1 |
| **Alderuccio et al.** [78] | 2017 | CASE REPORT | 1 |
| **Garcìa et al.** [79] | 2017 | CASE REPORT | 1 |
| **Johnson et al.** [80] | 2017 | CASE SERIES | 18 |
| **Nogueira Batista et al.** [81] | 2017 | CASE REPORT | 1 |
| **Patzelt et al.** [82] | 2017 | CASE REPORT | 1 |
| **Dashevsky et al.** [83] | 2018 | CASE SERIES | 11 |
| **Kricheldorff et al.** [84] | 2018 | CASE SERIES | 6 |
| **Ronchi et al.** [85] | 2018 | CASE SERIES | 1 |
| **Becherer et al.** [39] | 2019 | SYSTEMATIC REVIEW | 17 |
| **D’Alessandris et al.** [86] | 2019 | CASE REPORT | 1 |
| **Fricke et al.** [87] | 2019 | CASE REPORT | 1 |
| **Har-Shai et al.** [41] | 2019 | CASE REPORT | 1 |
| **Campanale et al.** [9] | 2020 | SYSTEMATIC REVIEW | 46 |
| **Evans et al.** [88] | 2020 | CASE REPORT | 1 |
| **Kim et al.** [89] | 2020 | CASE REPORT | 1 |
| **Laurent et al.** [22] | 2020 | SYSTEMATIC REVIEW | 34 |
| **Lee et al.** [90] | 2020 | CASE REPORT | 1 |
| **Misad et al.** [91] | 2020 | CASE REPORT | 1 |
| **Nelson et al.** [23] | 2020 | SYSTEMATIC REVIEW | 11 |
| **Ohishi et al.** [36] | 2020 | CASE REPORT | 1 |
| **Thienpaitoon et al.** [92] | 2020 | CASE REPORT | 1 |
| **Everest et al.** [1] | 2021 | CASE REPORT | 1 |
| **Barnea et al.** [21] | 2022 | CASE REPORT | 1 |
